# Myeloid Sarcoma after Allogenic Stem Cell Transplantation for Acute Myeloid Leukemia: Successful Consolidation Treatment Approaches in Two Patients

**DOI:** 10.1155/2018/7697283

**Published:** 2018-02-28

**Authors:** Silje Johansen, Hilde Kollsete Gjelberg, Aymen Bushra Ahmed, Øystein Bruserud, Håkon Reikvam

**Affiliations:** ^1^Section of Hematology, Department of Medicine, Haukeland University Hospital, 5021 Bergen, Norway; ^2^Department of Pathology, Haukeland University Hospital, 5021 Bergen, Norway; ^3^Department of Clinical Science, University of Bergen, 5021 Bergen, Norway

## Abstract

Myeloid sarcoma is an extramedullary (EM) manifestation (i.e., manifestation outside the bone marrow) of acute myeloid leukemia (AML); it is assumed to be relatively uncommon and can be the only manifestation of leukemia relapse after allogenic stem cell transplantation (allo-SCT). An EM sarcoma can manifest in any part of the body, although preferentially manifesting in immunological sanctuary sites as a single or multiple tumors. The development of myeloid sarcoma after allo-SCT is associated with certain cytogenetic abnormalities, developing of graft versus host disease (GVHD), and treatment with donor lymphocytes infusion (DLI). It is believed that posttransplant myeloid sarcomas develop because the EM sites evade immune surveillance. We present two patients with EM myeloid sarcoma in the breast and epipharynx, respectively, as the only manifestation of leukemia relapse. Both patients were treated with a combination of local and systemic therapy, with successfully longtime disease-free survival. Based on these two case reports, we give an updated review of the literature and discuss the pathogenesis, diagnosis, and treatment of EM sarcoma as the only manifestation of AML relapse after allo-SCT. There are no standard guidelines for the treatment of myeloid sarcomas in allotransplant recipients. In our opinion, the treatment of these patients needs to be individualized and should include local treatment (i.e., radiotherapy) combined with systemic therapy (i.e., chemotherapy, immunotherapy, DLI, or retransplantation). The treatment has to consider both the need for sufficient antileukemic efficiency versus the risk of severe complications due to cumulative toxicity.

## 1. Introduction

Myeloid sarcoma is a rare manifestation of AML and can appear concomitantly with, following or rarely antedating the onset of bone marrow leukemia [1]. MS can be the sole manifest of AML relapse after allo-SCT. We describe two patients presenting with myeloid sarcoma as the only sign of AML relapse after allo-SCT. We discuss diagnostic and therapeutic aspects of this assumed rare manifestation of relapse after allo-SCT.

## 2. Patients

### 2.1. Patient 1

**Figure 1:**
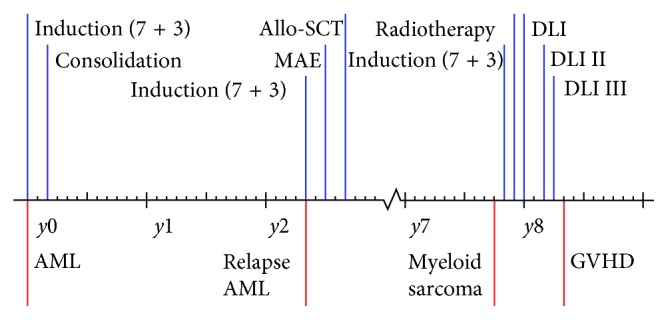
Timeframe regarding diagnosis and treatment of patient 1. The figure presents the main treatment features and therapeutic approaches in patient 1.

The patient was a 40-year-old Asian female diagnosed with AML during pregnancy (Figure 1). The bone marrow examination then showed 90% myeloblasts, with immunophenotype CD45dim/117+/13dim/33+/56−/2−/15−/14−/11b−/99+/HLA-DR−; CD34 positivity was detected for a subpopulation of 8% of blasts. Karyotyping showed t(7;11) as the only cytogenetic abnormality; this translocation between chromosomes 7p15 and 11p15 involved the *NUP98* gene on chromosome 11. Medical termination was performed before she received conventional induction therapy with daunorubicin 50 mg/m^2^ once daily on days 1–3 and cytarabine 200 mg/m^2^ as daily continuous infusion on days 1–7, and she reached complete hematological remission after this single induction cycle. This treatment was followed by consolidation therapy with 4 cycles of high-dose cytarabine, each of these cycles consisting of cytarabine 3 g/m^2^ twice daily on days 1, 3, and 5.

Relapsed disease was diagnosed 24 months after the first treatment was completed; at the time of relapse, she was pregnant in the 28th week and she was induced for labor in the 29th week. Cytogenetic analysis detected the original t(7;11) translocation in 6 out of 14 metaphases, and additional t(12;17) with a translocation between chromosome 12p11 and 17q11 was also detected together with loss of the derivative chromosome 17. The latter abnormality leads to the loss of genes on 17p including the *p53* gene. The immunophenotypic features were similar as at initial diagnosis. New induction treatment was performed by idarubicin 12 mg/m^2^ once daily on days 1–3 and cytarabine 200 mg/m^2^ as daily continuous infusion on days 1–7, and again she reached complete hematological remission after one induction cycle. Then she was given further consolidation therapy with one cycle of amsakrin 150 mg/m^2^ once daily on days 1–5, etoposide 110 mg/m^2^ once daily on days 1–5, and cytarabine 200 mg/m^2^ as daily continuous infusion on days 1–5. After this, she proceeded to myeloablative conditioning (MAC) allo-SCT with a matched sibling donor. She showed no sign of GVHD at any time after the transplantation, and she achieved full donor chimerism.

**Figure 2:**
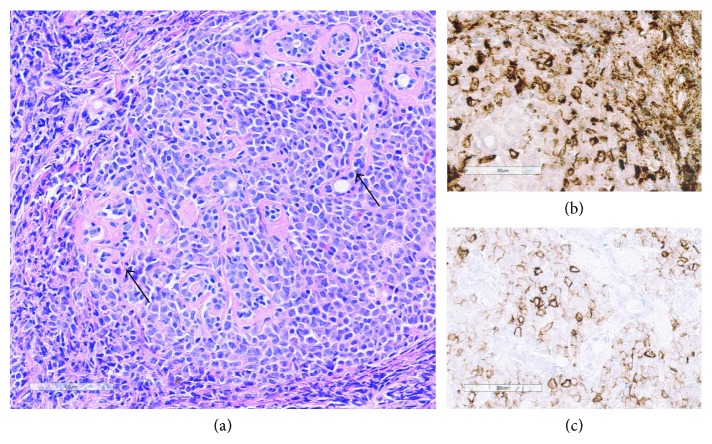
Stained biopsy from patient 1. (a) Histologic image (hematoxylin/eosin staining) of the breast biopsy showing a diffuse infiltrate of tumor cells with scant cytoplasm and large nuclei with “open” chromatin pattern and distinct nucleoli, consistent with blasts. The blasts infiltrate around small residual acini (black arrows) in a lobule (white arrow). (b) The tumor cells are myeloperoxidase positive by immunohistochemistry. (c) Dim to moderate positivity by immunohistochemical staining for CD56. Size bars: 80 *µ*m.

She presented with a tumor in her left breast 62 months after the allo-SCT, and biopsy confirmed the diagnosis of myeloid sarcoma (Figure 2). Immunophenotypic features were compatible with AML clone found at diagnosis and first relapse, although CD56 was dim positive. Positron emission tomography (PET) scan showed increased uptake in the tumor as well as two smaller lesions in the right breast. Bone marrow examination showed no evidence of AML by morphological and flow cytometric examination. She received induction treatment by idarubicin 12 mg/m^2^ once daily on days 1–3 and cytarabine 200 mg/m^2^ as daily continuous infusion on days 1–7. Control PET examination after one induction cycle showed complete regression of fluorodeoxyglucose (FDG) uptake in the left breast. The patient denied further intensive chemotherapy and retransplantation. She therefore received consolidation treatment by radiation therapy 2 Gy × 15 (total dose 30 Gy) against both breasts. In addition, she received three donor lymphocyte infusions (DLIs) in total 1.6 × 10^8^ cells/kg; G-CSF-primed cells harvested at the time of stem cell harvesting were used for all three DLIs. After the last DLI, she presented with oral and skin manifestations consistent with GVHD and this diagnosis was confirmed with biopsy/histology. She has now been in complete remission without any signs of leukemia for 22 months after diagnosis of relapse as MS.

### 2.2. Patient 2

**Figure 3:**
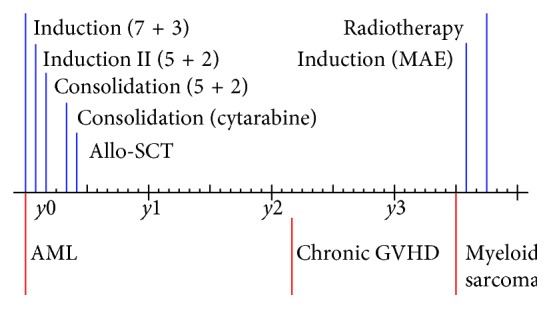
Timeframe regarding diagnostic and treatment of patient 2. The figure presents the main treatment features and therapeutic approaches in patient 2.

**Figure 4:**
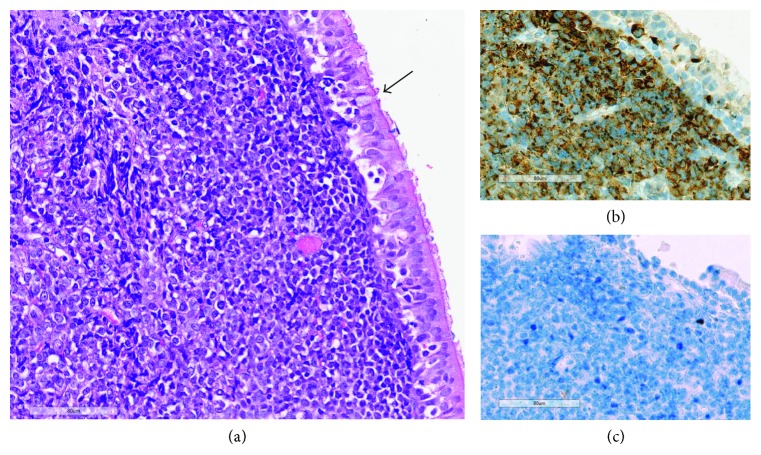
Stained biopsy from patient 2. (a) Subepithelial diffuse infiltrate of blasts in the biopsy specimen from the epipharyngeal lesion (hematoxylin/eosin staining). Black arrow: respiratory epithelium. (b) Virtually all the blasts are myeloperoxidase positive by immunohistochemistry. (c) The tumor cells are negative for CD56. Size bars: 80 *µ*m.

This patient was a 34-year-old Caucasian male diagnosed with normal karyotype AML (46, XY) without mutations in the *FLT3* or *NPM1* genes (Figure 3). With multiparameter flow cytometric analysis, the immunophenotype was described as CD7+/CD11c+/CD13+/CD14−/CD15−/CD33+/CD34+/CD117+/HLA-DR+. CD56 was positive only for 3% of blast cells. The first induction cycle included cytarabine plus an anthracycline (idarubicin 12 mg/m^2^ once daily on days 1–3 and cytarabine 200 mg/m^2^ as daily continuous infusion on days 1–7). However, he had persistent leukemic blasts in the bone marrow 16 days after start of this cycle; additional treatment with cytarabine and idarubicin (idarubicin 12 mg/m^2^ once daily on days 1 and 2; cytarabine 200 mg/m^2^ as daily continuous infusion on days 1–5) was started the next day. He now reached a complete hematological remission and received consolidation with another cycle of cytarabine plus idarubicin (5 + 2 regimen) and thereafter one cycle of high-dose cytarabine. The patient proceeded to allo-SCT with myeloablative conditioning based on busulfan plus cyclophosphamide with an HLA-matched sibling donor. 21 months after the transplantation, he presented with increased liver enzymes and histological changes in his oral cavity confirmed by biopsy to be chronic GVHD (cGVHD). Thirty-nine months after allo-SCT, he presented with symptoms from his left ear, and he was diagnosed with an epipharyngeal tumor; biopsies from this lesion demonstrated tumor of myeloid origin (Figure 4). The tumor cells stained negative for CD56 with immunohistochemistry. His bone marrow showed normal cellularity with no signs of leukemia. Thus, he had MS as the only manifestation of posttransplant AML relapse. He received induction treatment with amsakrin 150 mg/m^2^ once daily on days 1–5, etoposide 110 mg/m^2^ once daily on days 1–5, and cytarabine 200 mg/m^2^ as daily continuous infusion on days 1–5; peripheral blood and bone marrow examination were consistent with complete hematological remission, and MRI showed regress of the epipharyngeal tumor. The consolidation treatment was radiotherapy with 2 Gy × 20 (totally 40 Gy). After this treatment, he developed mucosal skin lesions in mouth consistent with cGVHD; for this reason, he never received DLI. MRI taken after completion of radiotherapy showed total regress of the tumor. He is still in complete remission 72 months after the diagnosis of relapse.

## 3. Discussion

MS is a tumor of immature myeloid cells located at an extramedullary (EM) site; it can develop in any organ or site in the body but is more common at the immunological sanctuary sites of the testis, ovary, and central nervous system (CNS) [2, 3]. Results from a multicenter survey implies skin and lymph nodes as frequently involved in *de novo* MS, whereas soft tissues are more often involved in secondary MS [3]. The involvement of breast is uncommon [4], although reported with relapsed disease [5]. Thus, both our patients with posttransplant MS had uncommon localizations.

An EM AML relapse will usually progress to involve other EM sites as well as the bone marrow within one year [2]. The incidence of EM AML relapse after allo-SCT of AML was 0.65% in a large retrospective study from the European Group of Blood and Marrow Transplantation (EBMT) [6]. However, recent studies indicate that the incidence is higher, and posttransplant EM relapse has been described in 5–12% of allotransplanted AML patients, accounting for 7–46% of all AML relapses [2, 7, 8]. Among longtime survivals, the incidence has been reported to be as high as 20% [2, 9], and EM relapse has been described until several years after transplantation [2, 5]. The diagnosis is often delayed and the relapse is usually diagnosed when it becomes symptomatic, because no standardized strategy for the surveillance to detect posttransplant EM relapse exists [7]. Measurable residual disease (MRD), either by multiparameter flow cytometry or by genetic molecular markers, is established as an independent prognostic marker in AML [10]. However, the methodology of these approaches has currently not been qualitatively or quantitatively standardized, making their use in clinical practice challenging [10], and none of our patients was followed by MRD monitoring. Furthermore, if detecting of MRD could proceed, diagnosis of EM AML relapse remains elusive, although should be further investigated in upcoming clinical trials.

The best treatment of isolated EM relapse after allo-SCT is unknown [8, 11]. Previous induction and conditioning treatment causing cumulative toxicities and high-dose chemotherapy with suppression of the graft versus leukemia (GVL) reactivity have to be considered when deciding a therapeutic strategy with sufficient antileukemic efficiency versus the risk of severe and unacceptable toxicity [8, 11]. The prognosis of solitary EM relapse remains poor, although it seems slightly better than bone marrow relapse alone or combined bone marrow and EM relapse [7].

It is believed that the general immune-mediated antileukemic reactivity mediated by the general graft versus host reactivity that is associated with the occurrence of GVHD preferentially maintains bone marrow remission but does not prevent EM relapse [8]. The occurrence of acute GVHD (aGVHD) is significantly associated with better bone marrow relapse-free survival; however, the EM relapse rate for patients with or without a GVHD seems to be similar [7]. There are several possible explanations for this phenomenon. Firstly, AML cells in peripheral tissues may evade immune surveillance [2], as the number of effector cells for immune-mediated antileukemic reactivity is higher in the bone marrow. The partial loss of several human leukocyte antigen (HLA) class I genes may further decrease the general efficiency of the antileukemic immune reactivity [7]. Secondly, the CD56 expression in AML cells mediates cell-cell adhesion and is highly expressed in various tissues; these adhesion molecules seem to be involved in EM homing and may then support EM AML cell survival [7]. It has been suggested that the CD56 cell surface expression together with the encoded fusion proteins in patients with the chromosomal abnormalities t(8:21) and inv(16) is associated with EM AML cell infiltration [7], although it is unknown whether these mechanisms are important for posttransplant EM relapse [7]. EM presentation has also been associated with other chromosomal abnormalities like trisomy of chromosomes 4, 8, and 11 as well as deletion of chromosomes 5q, 16q, and 20q [12]. Noteworthy, the myoblast for one of our patients was only dim positive for CD56 in the biopsy of the relapsed EM AML in the breast, and for the other patient only 3% of the blast cells at the time of AML diagnosis was CD56 positive. Thirdly, some studies suggest that EM relapse is higher after allo-SCT compared to auto-SCT and that the incidence of MS relapse seems to be increased after DLI and in retransplanted patients [7, 13–15]. Other factors associated with increased risk of EM after allotransplantation are relapsed or refractory disease at time of transplant, unfavorable cytogenetics, EM disease before allo-SCT, retransplantation, age below 18 years, and FAB subtypes M4/M5 [7, 12]. Finally, if certain pretransplant conditioning regimens are more effective with regard to prevention of posttransplant EM relapse is unknown [7]. None of these risk factors were present in our patients. Both patients were in complete hematological remission at the time of transplantation, they received busulfan-based conditioning, and one of them had clinical signs of GVHD before EM relapse was diagnosed.

There are no established guidelines for the treatment of EM relapse after allo-SCT. The common practice is a combination of local and systemic treatment including intensive chemotherapy, local radiotherapy, DLI, and/or retransplantation [2, 9]. Anti-AML therapy seems to be effective in patients presenting with MS alone [1], and systemic chemotherapy is required rather than surgery or radiotherapy alone to prevent relapse and disease progression. However, many of these previous studies do not differentiate between *de novo* and secondary MS with regard to treatment and prognosis [3], and treatment approaches for EM relapse after allo-SCT should probably involve combination of local and systemic therapy to prevent systemic relapse [7, 16].

Treatment options for patients with EM relapse should be considered individually [17]. Since radiological examination after induction treatment did not reveal disease activity in the breasts for our first patient, further surgery was therefore not performed. DLI is regarded as an effective treatment of posttransplant bone marrow relapse, although may not be equally effective for EM relapse [2, 18]. However, the combination of DLI with chemotherapy will often make it difficult to differentiate the effects of the immunotherapy and the chemotherapy [2]. Only one of our patients received DLI, although discontinued after three infusions because of GVHD. The curative effect of allo-SCT in AML is considered to be due to a combined effect of conditioning chemotherapy and posttransplant immune-mediated antileukemic activity, and for this reason it is probably important to maintain this immune reactivity in patients with EM relapse [8].

A possible treatment for isolated EM relapse is gemtuzumab/ozogamicin (GO), acting by depleting CD33-expressing leukemic cells and possible bolstering antileukemic immune reactivity [8, 19]. The risk of severe toxicity and especially hepatic injury with veno-occlusive disease (VOD) should be taken into account [8]. Another systemic active agent that does not abrogate the antileukemic immune reactivity is the hypomethylating agents 5-azacitidine and decitabine, which even might enhance the GVL effect. These agents induce AML cell differentiation and increase the expression of HLA antigens as well as tumor-associated antigens [7]; this strategy is used for the treatment of posttransplant bone marrow relapse of both AML and myelodysplastic syndromes (MDS) [7, 9]. Case reports suggest that these agents may be effective even for patients with posttransplant EM and previous treatment failure after DLI, radiotherapy, and intensive chemotherapy [9, 20]. Finally, CD56 expression in leukemic cells and antibodies conjugated with a toxin or a radioisotope may represent future strategies for the treatment of these patients [7].

## 4. Conclusion

EM posttransplant AML relapse may be more common than previously assumed, and its prognosis remains poor even though possibly slightly better than for combined EM and bone marrow relapse or bone marrow relapse alone. No guidelines for standard treatment of these patients are available, but a common practice is systemic chemotherapy or immunotherapy (i.e., DLI or retransplantation) combined with local radiotherapy. Cumulative toxicities due to previous chemotherapy and the risk of suppressing clinically relevant antileukemic immune reactivity have to be considered when the treatment of such patients is decided. Our two patients received intensive chemotherapy induction followed by consolidating radiotherapy, and only one of them received DLI; both patients are still alive without relapse. Future studies should focus on the development of diagnostic strategies for earlier detection of EM relapse and the identification of molecular mechanisms (i.e., possible therapeutic targets) behind EM homing of leukemic cells.
